# Analysis of Olfactive Prints from Artificial Lung Cancer Volatolome with Nanocomposite-Based vQRS Arrays for Healthcare

**DOI:** 10.3390/bios15110742

**Published:** 2025-11-04

**Authors:** Abhishek Sachan, Mickaël Castro, Jean-François Feller

**Affiliations:** Smart Plastics Group, IRDL CNRS 6027, University of South Britanny (UBS), 56100 Lorient, Francemickael.castro@univ-ubs.fr (M.C.)

**Keywords:** breath analysis, e-nose, arrays, quantum resistive vapor sensors (vQRSs), chemo-resistive, nanocomposites, spray layer by layer (sLbL), nano-assembly, disease diagnosis

## Abstract

Exhaled breath analysis is emerging as one of the most promising non-invasive strategies for the early detection of life-threatening diseases, especially lung cancer, where rapid and reliable diagnosis remains a major clinical challenge. In this study, we designed and optimized an electronic nose (e-nose) platform composed of quantum resistive vapor sensors (vQRSs) engineered by polymer-carbon nanotube nanocomposites via spray layer-by-layer assembly. Each sensor was tailored through specific polymer functionalization to tune selectivity and enhance sensitivity toward volatile organic compounds (VOCs) of medical relevance. The sensor array, combined with linear discriminant analysis (LDA), demonstrated the ability to accurately discriminate between cancer-related biomarkers in synthetic blends, even when present at trace concentrations within complex volatile backgrounds. Beyond artificial mixtures, the system successfully distinguished real exhaled breath samples collected under challenging conditions, including before and after smoking and alcohol consumption. These results not only validate the robustness and reproducibility of the vQRS-based array but also highlight its potential as a versatile diagnostic tool. Overall, this work underscores the relevance of nanocomposite chemo-resistive arrays for breathomics and paves the way for their integration into future portable e-nose devices dedicated to telemedicine, continuous monitoring, and early-stage disease diagnosis.

## 1. Introduction

**Figure 1:**
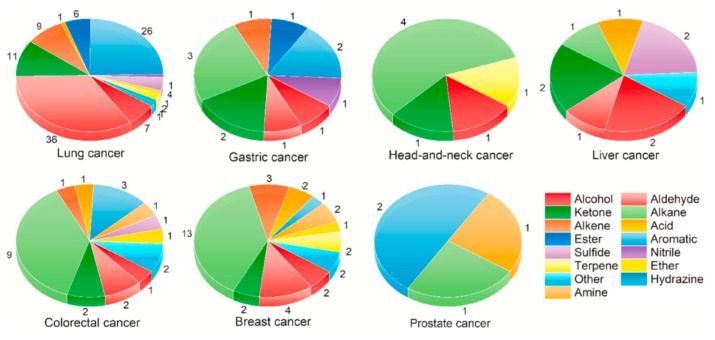
Summary of some cancer volatile biomarkers from urine [24].

Human exhaled breath analysis is an effective method for the early diagnosis of various fatal diseases [1] and has been gaining popularity over the past ten years. This approach was first introduced by Pauling et al. [2], who detected more than two hundred VOCs in human breath. Some of these VOCs have been identified as disease biomarkers, sometimes specific to certain pathologies. Precise detection of VOCs in exhaled breath can lead to early-stage identification of diseases and could potentially replace or precede existing clinical diagnostic tools. Human exhaled breath provides information about the condition and health of the body [3,4,5]. The detection of biomarkers in exhaled breath remains a challenge at the clinical level, but it offers one of the simplest methods for the early detection of lung cancer [6,7,8,9,10]. It has many advantages, including low cost, painless procedures, ease of operation, repeatability, and most importantly, non-invasiveness [11,12,13,14], i.e., gas-phase testing is easier than the examination of biological tissue or blood [15,16]. Most VOCs present in breath are exogenous, meaning that they result from environmental exposure. For diagnostic purposes, endogenous VOCs (produced as by-products of the body’s metabolic activities, such as pentane, isoprene, ketones, aldehydes, mercaptans, and amines) have been listed as important targets [5]. However, exhaled breath contains very low amounts of these biomarkers, typically at ppb or ppt levels [17]. This low concentration range depends on the body’s condition, as sick individuals generally show higher biomarker levels compared to healthy ones. In the literature, several biomarkers have been identified as related to specific diseases, some of which are listed in Figure 1 [18,19,20,21,22,23,24]. For instance, diabetes patients have acetonic breath [25,26], toluene is a biomarker for lung cancer [27,28,29,30,31], ammonia and nitrogen oxide are related to kidney failure [32,33] and asthma [34,35], respectively, ascorbic acid (AA) and uric acid (UA) are biomarkers of Parkinson’s and Alzheimer’s disease [36,37], and finally, heart disease increases the level of pentane in the breath [38]. Among these diseases, lung cancer is one of the most fatal, causing millions of deaths each year. One remaining challenge is the difficulty of detecting its initial stages with current diagnostic tools, whereas early detection could save 60–80% of lung cancer patients. Presently, only about 15% of patients are diagnosed at an early stage [39,40,41], enabling appropriate care. In addition, current methods are sometimes painful, invasive, and unreliable, strengthening the idea that volatolomics is a relevant tool for lung cancer detection through exhaled breath analysis [42,43,44,45,46,47,48] or even urine volatiles [24].

The analysis of breath can be performed using chemo-resistive, conducting polymer nanocomposite-based sensors. This type of sensor, made of an electrically conducting nanocarbon architecture functionalized and stabilized within an amorphous polymer matrix, has been widely studied for vapor and gas sensing. The performance of these sensors, including their sensitivity and selectivity towards specific vapors, has been continuously improved by researchers [49,50,51,52,53,54,55].

Quantum resistive vapor sensors (vQRSs) are synthesized by dispersing nanocarbons within various polymer matrices. These polymers simultaneously impart selectivity toward the target analytes and stabilize the conductive architecture responsible for the chemo-resistive response. Consequently, the resulting conductive polymer nanocomposite (CPC)-based transducers can be readily tailored in terms of molecular interactions, predominantly governed by van der Waals forces. vQRSs are based on multilayered transducers composed of conductive polymer nanocomposites obtained through the solution-phase assembly of carbon nanofillers—such as carbon nanotubes [56], graphene [57], fullerenes [58], carbon nano-onions [59], and their possible hybrids [60]—with various polymers. The resulting conductive network forms a random architecture comprising billions of interconnections that can be disrupted by the diffusion of only a few parts per million of volatile compounds through the transducer. The disruption of carbon–carbon junctions induces a pronounced change in electrical resistance, as charge transport in these materials is governed by quantum tunneling (an effect exponentially dependent on the inter-filler distance). Even a separation of a few nanometres significantly alters the energy required for electrons to tunnel across the gap. This tunneling-based conduction mechanism is the origin of the sensor’s name. Furthermore, the nano-assembly process employed (spray layer-by-layer deposition (sLbL)) enables precise tuning of the electrical resistance and offers fine control over the structural organization from the nanoscale to the microscale. Owing to their mechanical compliance, these transducers can be deposited onto flexible substrates, including textiles, which is particularly advantageous for wearable electronic applications. In addition, CPC-based transducers do not require heating for moisture desorption, unlike most metal oxide sensors. As a result, their power consumption remains very low, paving the way for autonomous electronic noses powered by printable photovoltaic or piezoelectric energy-harvesting devices.

As breath is a very complex blend of dozens of volatile biomarkers, a set of chemo-resistive sensors with different selectivities towards VOCs of interest is therefore required for a complete analysis. This set of sensors is usually referred to as an electronic nose or e-nose. The data obtained by the e-nose can be analyzed with different statistical methods such as principal component analysis (PCA), linear discriminant analysis (LDA), and artificial neural networks (ANNs) [61,62,63,64,65,66,67,68,69,70,71]. The aim of processing the chemo-resistive responses is to provide a “breath print” of each human exhaled sample to allow precise classification. Finally, after performing the learning step of the e-nose, a proper algorithm must distinguish healthy from cancerous breath prints [72,73,74,75,76,77,78].

In the present study, an e-nose was assembled using nine different optimized nanocomposite sensors. The selectivity of each sensor was modified by the non-covalent functionalization of carbon nanotubes with different polymer matrices of complementary chemical properties during formulation. The selected sensors were then exposed to the targeted biomarker VOC present in blends as well as to exhaled breath samples. Biomarker concentrations (ppm) were varied across low, medium, and high levels in a background composed of several other VOCs. Up to three biomarkers were mixed in the background to observe the ability of the e-nose to discriminate target molecules from interfering compounds. In the second part of this study, the same e-nose was tested with real human exhaled breath collected under different environmental conditions, i.e., before and after smoking, as well as before and after alcohol consumption. The exhaled breath tests were repeated to evaluate the reproducibility of the results.

## 2. Experimental

### 2.1. Materials

NC7000 multiwalled carbon nanotubes (CNTs) were kindly provided by Nanocyl S A, (Sambreville, Belgium). CNTs were produced by a catalytic carbon vapor deposition (CCVD) method. The NC700 grade has a purity of 90%, an average diameter of 9.5 nm and an average length of 1.5 µm. CNTs were used as received without any further purification. Poly(styrene-co-[propyl methacryl hepta isobutyl]), abbreviated PS-co-POSS, and Poly(methyl methacrylate-co-[propyl methacryl hepta isobutyl]), abbreviated PMMA-co-POSS, were procured from Sigma-Aldrich (Evry, France). Both random copolymers contain 45 wt.% of POSS. Poly(caprolactone), PCL, had an average molar mass in number of M_n_ = 80,000 g·mol^−1^, poly(ethylene glycol), PEG, had an average molar mass in number of M_n_ = 10,000 g·mol^−1^, and poly(methyl methacrylate), PMMA, had an average molar mass in weight of M_w_ = 120,000 g·mol^−1^. These polymers were purchased from Sigma-Aldrich, France. Poly(vinyl pyrrolidone), PVP, had an average molar mass in weight of M_w_ = 1,300,000 g·mol^−1^, and poly(styrene), PS, had an average molar mass in weight of M_w_ = 250,000 g·mol^−1^. These polymers were obtained from Acros Organics (Illkirch, France). Poly(lactic acid), PLA, had a melt flow index range of MFI = 15–30 g·10 min^−1^, and poly(styrene-co-acrylonitrile), SAN, had a molar composition of 75% styrene and 25% acrylonitrile. They were obtained from NatureWorks (Naarden, The Netherlands) and PolyScience Inc. (Issy les Moulineaux, France), respectively. All solvents used in the blends (chloroform, ethanol, propanol, toluene, xylene, octane, trimethyl benzene (TMB) and benzaldehyde) and for calibration (propanol, ethanol, methanol, acetone, toluene, xylene, 2 ethyl 1 hexanol, cyclohexanone, hexene, 2 methyl 1 propanol, isopropanol) were purchased from Sigma-Aldrich and used as such without any further purification. Tedlar^®^ bags (Kiel, Germany) for gas sampling (2 dm^3^ volume) with septum and screw cap valve were also purchased by Sigma-Aldrich (Evry, France) for VOC blend preparation and exhaled breath collection.

### 2.2. Transducers’ Nano-Assembly by sLbL

From the above materials, vQRSs were fabricated by spraying layer-by-layer (sLbL) dispersions, prepared by adding 2 wt.% of CNTs to the polymer (i.e., 200 mg of CPC) in 20 cm^3^ of chloroform under ultrasonication for 6 h at 50 °C using a Branson 3510 operating at 100 W and 40 kHz. Nine different polymers were used. Four layers of each were sprayed over home-made interdigitated electrodes (IDEs) composed of Ag (25%)/Pd (75%) tracks, separated by a 15 µm ceramic gap, and prepared by cleaving 22 nF capacitors. The electrodes were polished and then cleaned with ethanol to remove any surface contamination.

The sLbL device consisted of a nozzle mounted on a 3D printer frame to control x/y movement, operated with a Valve Mate 8040 (Nordson, Westlake, OH, USA) at a scan speed of Vs = 50 mm·s^−1^, a flow rate index of 2, a target-to-nozzle distance of 8 cm, and an air pressure of ps = 0.1 MPa. After fabrication, vQRSs were conditioned at 30 °C in a controlled atmosphere overnight. The initial resistance (R_0_) and the composition of the sensors are given in Table 1. The e-nose was assembled from the nine sensors listed in Table 1.

### 2.3. Transducers’ Calibration

**Figure 2:**
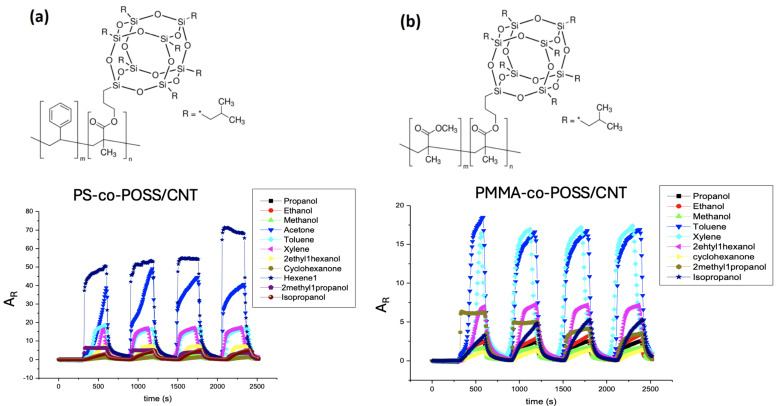
Example of (**a**) PS-co-POSS/CNT and (**b**) PMMA-co-POSS/CNT sensors’ chemo-resistive responses (A_R_) to the set of calibration vapors.

The nine different transducers assembled into the e-nose were selected thanks to their affinity to a set of calibration vapors (propanol, ethanol, methanol, acetone, toluene, xylene, 2 ethyl 1 hexanol, cyclohexanone, hexene, 2 methyl 1 propanol, isopropanol) chosen for their differences in hydrophilic/hydrophobic balance and nature of interactions evaluated by their Flory Huggins c_12_ parameter, as already detailed in previous study [79,80]. Figure 2 shows typical responses of two transducers, i.e., PS-co-POSS/CNT and PMMA-co-POSS/CNT, when exposed to calibration vapors. The response times are very quick, usually less than one second, and the baseline shows minimal drift. The amplitude (A_R_) defined in Equation (1) can be very large (more than 60 in the case of PS-co-POSS/CNT exposed to 1-hexene or acetone), sometimes compromising reproducibility.

(1) $$A_{R}=\frac{R-R_{0}}{R_{0}}$$

where R_0_ is initial resistance of sensors under a dry nitrogen stream, and R is the resistance measured in the presence of the bag output.

However, in most cases, when A_R_ was less than 20, the signals were very reproducible and stable, despite the large number of VOCs delivered to the CPC transducers. After sorption, vQRSs returned to their initial resistance (R_0_) upon desorption, which suggests that almost all molecules were released from the nanocomposite sensitive films. Nevertheless, some cycles were atypical and were therefore excluded from the calculation of the average chemo-resistive amplitude. In the following part of the study, the VOC concentrations used were hundreds of times lower than under saturated conditions (i.e., from several hundred to a thousand ppm), and thus, all responses were below 5 and reproducible.

Once the sensitivity of all sensors chosen to integrate the e-nose is checked, the device can be exposed to the different samples of breath, artificial and human.

### 2.4. Artificial Breath Samples’ Preparation

**Figure 3:**
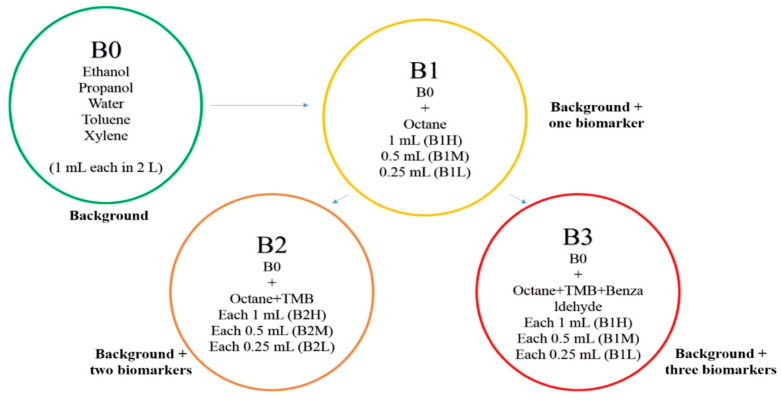
Preparation of blends and their variations for e-nose analysis.

VOC blends were prepared using Tedlar^®^ gas sampling bags. The bags were equipped with screw-cap valves and septa. The desired number of VOCs was injected through the septum in the bag. After adding all VOCs in a particular blend, the bag was flushed with dry air. Blown bags were given some time to reach equilibrium. Four different blends were prepared. The first one was background blend (B_0_) including water, ethanol, propanol, toluene, and xylene. A total of 1 cm^3^ of each was injected into the bag, and at room temperature, VOCs evaporated according to their partial pressure in the headspace. The second blend (B_1_) was prepared by adding one cancer biomarker (octane) to B_0_. Octane was added in three different volumes: 0.25 cm^3^ (low), 0.5 cm^3^ (medium), and 1 cm^3^ (high) to B_0_. In this case, the blends were named B_1_L, B_1_M, and B_1_H, respectively. The concentration variation was implemented to check the ability of the e-nose to detect low ppm of biomarkers in large numbers of background VOCs. Similarly, two other cancer biomarker blends were selected and called B_2_ and B_3_, containing, respectively, two (octane, TMB) and three biomarkers (octane, TMB, benzaldehyde) added to B_0_. Further concentration of biomarkers was increased to make low (0.25 cm^3^ of each), medium (0.5 cm^3^ of each), and high (1 cm^3^ of each) variations of B_2_ and B_3_, as shown in Figure 3.

Table 2 gives more details on VOC concentrations and designations in each blend.

The number of VOCs present inside the bag was calculated by the following Equation (2):

(2) $${\left[ppm\right]}_{blend}\;=\;{\left(mole\;fraction\right)}_{VOC}\;\times \;{\left[ppm\right]}_{saturated\;condition}$$

where *ppm_blend_* is the concentration (in ppm) of each vapor in each blend, the mole fraction of VOC represents the fraction of each vapor (in mole) in each blend, and *ppm_saturated condition_* is the concentration of each vapor at the saturated state.

### 2.5. Human Breath Samples’ Collection

Exhaled breath of two volunteers was collected in fresh Tedlar^®^ bags (Kiel, Germany). For the first volunteer (V_1_), samples were collected before and after smoking. This breath test was repeated three times within a single week. Exhaled breath from the second volunteer (V_2_) was collected before and after alcohol consumption, and this test was repeated twice. The exhaled breath was collected in the bag through a screw-cap valve. Specifically, alveolar breath was collected; i.e., volunteers were instructed to blow only the last part of their exhaled breath into the bag. Accordingly, both volunteers exhaled into the bag via a mouthpiece after a 5 s nasal exhalation. This process was repeated until the bag was filled nearly to its full capacity. Condensation of water and other VOCs was avoided by analysing all bags immediately after collection. All tests were conducted at room temperature (25 °C). First, the standardization of breath collection and analysis is crucial. However, it was underlined that the results of breath testing depend on several factors related to the method of sample collection, patients’ physiologic condition, and test environment [81]. The two volunteers were explained the ahead protocol of breath collection before obtaining their consent. Then, the protocol was validated by the Scientific and Ethical Committee of the University of South Brittany.

### 2.6. Breath Samples’ Analysis

**Figure 4:**
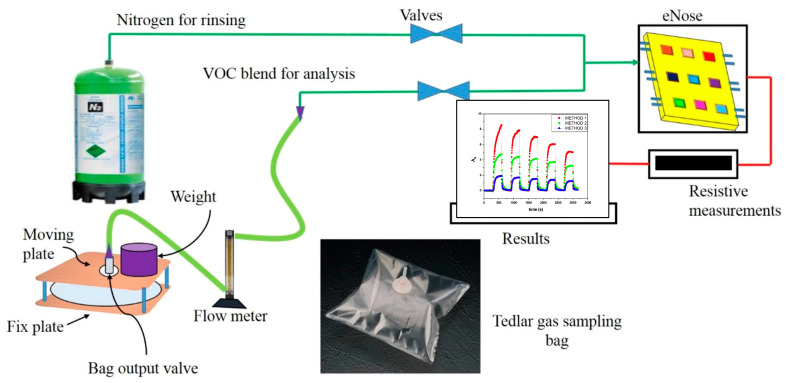
Tedlar^®^ bag and device to obtain a controlled output to the e-nose.

The Tedlar^®^ bags containing exhaled breath and VOC blend samples were characterized by analyzing the chemo-resistive responses of the e-nose (array of nine sensors). The testing cell is a rectangular cavity of 100 mm × 10 mm × 3 mm, in which the nine sensors are arranged in an array (e-nose). Dynamic testing of breath and VOC blends was performed using mass flow controllers, electrically controlled valves, and a homemade compression apparatus with a flow meter to ensure controlled output from the bags, as shown in Figure 4.

A Keithley 6517 (Tektronix Inc., Les Ulis, France) was used to measure the change in sensor resistance, and LabVIEW 2018 software was used to record the data. Alternate cycles of nitrogen at a flow rate of 500 cm^3^·min^−1^ were applied during the cleaning cycle, and bag output was set at 100 cm^3^·min^−1^ for 5 min cycle. Twelve cycles were tested for each bag. As expected, the electrical resistance of sensors was increased in presence of VOC or breath samples. Therefore, relative resistance of each cycle was calculated by Equation (1).

For each bag (blends and breath test), all the A_R_ values (for the nine sensors and twelve cycles each) were obtained and analyzed by a data analysis tool. The discrimination ability of e-nose was tested by the data analysis tool for a given set of cancer biomarker blends and breath samples.

### 2.7. Data Analysis: Feature Extraction

**Figure 5:**
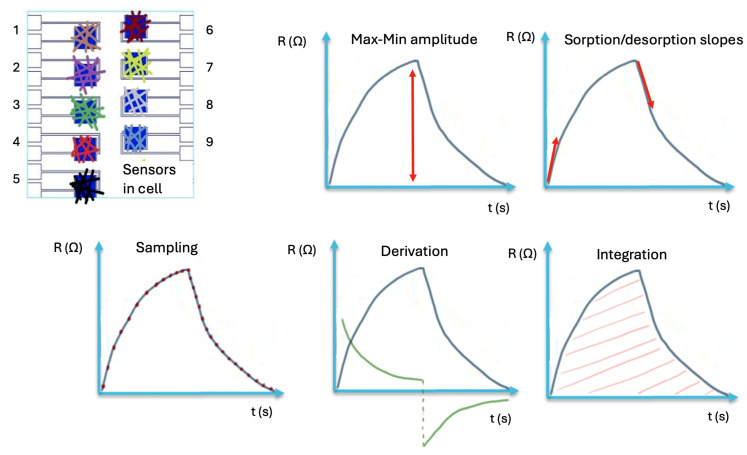
Example of feature extraction from sensors’ chemo-resistive signals: maximum amplitude, sorption/desoption slopes, sampling, derivation, integration [82].

During the gas/breath testing stage, the chemo-resistive responses are recorded, and the extracted features are assembled into a multidimensional matrix (sensors × cycles × gas/breath) for further analysis with classification algorithms. Different kinds of features can be extracted as described in Figure 5, such as amplitude, slopes, or area.

The primary features have a physical significance, such as signal amplitude and normalized sensors’ response. The secondary features, referred to as mapping features, are obtained from primary data, which can help to characterize the effectiveness of e-nose [61]. Both types of features are required for accurate interpretation of the e-nose data. From these features, the most effective ones (those associated with the lowest error rates) were selected for analysis using a pattern recognition algorithm. Pattern recognition represents the final step in e-nose data processing, allowing the classification of distinct olfactory prints.

In this study, the selected method was linear discriminant analysis (LDA), a form of discriminant function analysis (DFA) originally introduced by Fisher [83,84]. LDA is a supervised feature extraction technique that maintains a linear relationship with the original data points. By doing so, it minimizes within-group scattering of the data, thereby improving class separability. Here, the relative electrical responses (A_R_) from twelve successive cycles were processed with LDA to compare the different VOC blends and exhaled breath samples.

## 3. Results and Discussion

### 3.1. Analysis of VOC Blends

The sensor array (e-nose), composed of nine different sensors, was employed to detect and analyze various types of VOCs present in both synthetic blends and exhaled human breath. The selectivity of the transducers was tuned by the non-covalent functionalization of CNTs with polymer matrices of different chemical natures. The polymer-functionalized CNTs were then deposited using a layer-by-layer spraying (sLbL) process, forming a random conducting network less than 1 µm thick. In a previous study, Chatterjee et al. used six different transducers to detect eighteen VOCs under saturated conditions, demonstrating that PS, PLA, PCL, and PMMA are promising candidates for detecting a wide range of both polar and non-polar vapors [85]. Therefore, these four sensors were selected to integrate the array. PS- and PCL-based sensors were chosen for their ability to detect organic vapors such as toluene [86,87], whereas PMMA, PCL, and PLA were selected for their sensitivity to vapors such as ethanol, isopropanol, cyclohexane, and water [88]. PMMA was identified as a good candidate for the detection of alcohol and polar vapors [89]. Additional amorphous matrices, such as PVP, SAN, and PEG, were used to fabricate sensors sensitive to both polar and non-polar VOC molecules. Moreover, POSS copolymers of PS and PMMA were employed to enhance sensor sensitivity, in line with the findings of NAG et al. [90], who reported that the presence of POSS molecules at conducting junctions significantly increases the sensitivity of sensors, a result later confirmed by the work of Sachan et al. [80].

**Figure 6:**
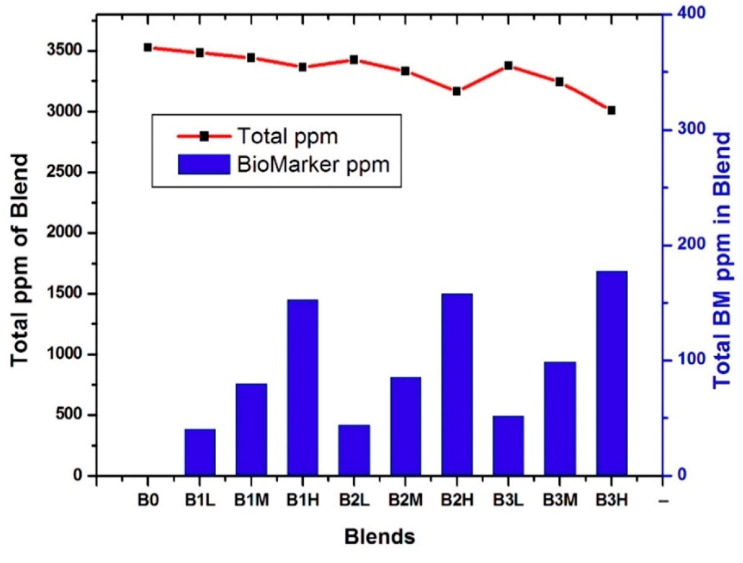
Overall concentration and lung cancer biomarkers (BM) concentration of each blend.

Figure 6 provides an overview of the different blends and their biomarker concentrations (in ppm). The average VOC concentration across all blends was 3367 ± 157 ppm. Within this large background of molecules (B_0_), the cancer biomarkers represented only a small fraction, decreasing from 5.8% to 3% and 1.5%, and were labeled as high, medium, and low in the respective blends. The e-nose was successively exposed to twelve cycles of each vapor blend, and A_R_ was recorded for each of the nine sensors and each cycle. The resulting data matrix thus contained (9 × 12 × 4) values, corresponding to the A_R_ of all sensors across twelve cycles and four blends, which were then used for LDA treatment.

**Figure 7:**
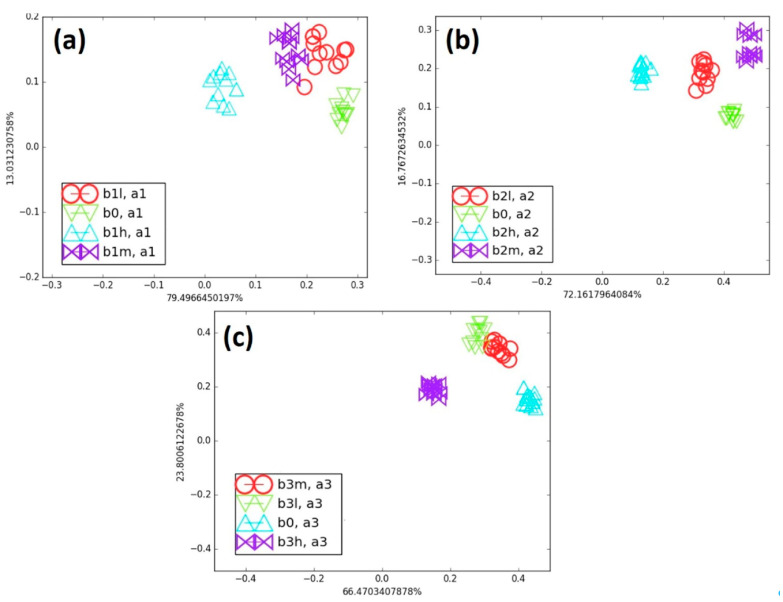
(**a**) Comparison of all B_1_ category blends with B_0_, (**b**) comparison of all B_2_ category blends with B_0_, and (**c**) comparison of all B_3_ category blends with B_0_ on LDA map.

Initially, each blend containing cancer biomarkers was individually compared with B_0_ on an LDA map. Figure 7 illustrates the influence of both the concentration of biomarkers and their number on clusters’ separation. For the B_1_ blends (Figure 7a), the clusters corresponding to B_1_H, B_1_M, and B_1_L were well separated from B_0_. The B_1_L data appears slightly scattered, as it contains only 40 ppm of octane within a 3500-ppm background. In contrast, B_1_M (80 ppm) and B_1_H (153 ppm) exhibit denser clusters. These results show that the e-nose was able to discriminate concentration variations of a single biomarker relative to B0. For the B_2_ blends (B_2_L, B_2_M, and B_2_H), a second biomarker (TMB) was added to 4, 8, and 15 ppm of octane. As shown in Figure 7b, the B_0_ data cluster remained nearly identical to the B_1_ case, but the addition of small amounts of TMB reduced the scattering of data points for each blend. The B_2_ clusters were clearly separated, confirming that the e-nose detected even slight changes in the blend composition. For the B_3_ blends, benzaldehyde was added together with octane and TMB and compared to B_0_ (Figure 7c). The B_3_ cluster was less scattered compared to those of B_1_ and B_2_. These results demonstrate that the successive addition of three volatile biomarkers densified the clusters without reducing the discrimination ability of the vQRS-based e-nose. Interestingly, increasing the biomarkers’ concentration in blends tended to shift the cluster closer to B_0_.

**Figure 8:**
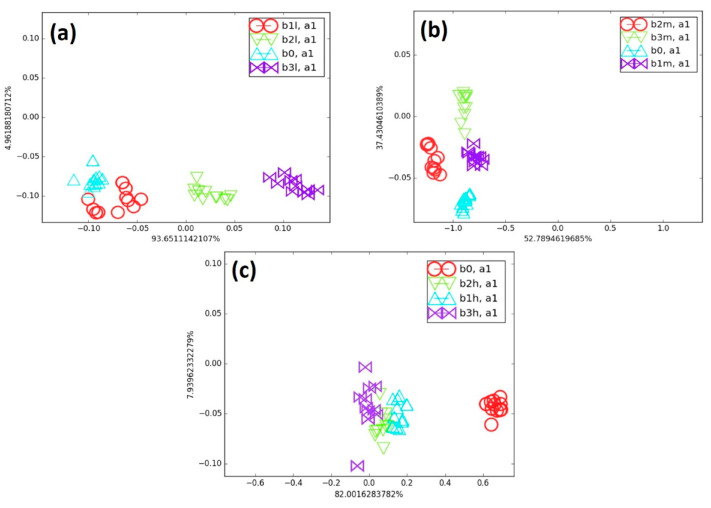
(**a**) Comparison of all low cancer biomarker blends with B_0_, (**b**) comparison of all medium cancer blends with B_0_, and (**c**) comparison of all high cancer blends with B_0_ on LDA map.

Figure 8 shows the effect of biomarker type at the same concentration on cluster discrimination. The blends were compared with B_0_ at low (L), medium (M), and high (H) concentrations of cancer biomarkers, which also makes it possible to assess the effect of biomarker number on the e-nose discrimination ability. For instance, in Figure 8a, the clusters of B_1_L, B_2_L, and B_3_L show increasing separation from B_0_ as their biomarker concentration rises from 41 to 44 and 52 ppm, respectively.

The total amount of biomarkers increased only slightly (from about 1 to 1.5% of the total concentration of the blend) as their number increased in B_0_. Since the change in A_R_ across all sensors collectively determined the position of the points on the LDA map, the presence of a higher number of biomarkers was clearly detected by the sensor array. Accordingly, the sensor responses shifted progressively, leading to a gradual separation from B_0_. In Figure 8b, all medium-concentration blends (B_1_M, B_2_M, and B_3_M) were well separated from the background (B_0_). In these blends, the biomarker concentrations were nearly twice those in the low-concentration blends, representing approximately 2.5 to 3% of the total blend. As biomarker concentration increased, the blend clusters shifted progressively, and their distance from B_0_ also increased. The high-concentration blends (B_1_H, B_2_H, and B_3_H) contained roughly three times more biomarkers than the low-concentration blends. All these high-concentration blends were clearly separated from B_0_ on the LDA map, as shown in Figure 8c. However, the e-nose encountered greater difficulty in differentiating the clusters of B_1_H, B_2_H, and B_3_H. At this concentration range, the A_R_ values (chemo-resistive responses) were nearly similar across all sensors, likely due to the faster saturation of the polymer matrix adsorption sites at such high biomarker content. Despite this limitation, the e-nose was still able to detect small amounts of cancer biomarkers in the presence of a large background of VOC and to effectively discriminate clusters in the low-concentration blends, using only one axis that accounted for nearly 94% of the total variance. Overall, the discrimination ability of the e-nose decreased as the biomarker concentration in the blends increased, as observed using the LDA algorithm.

### 3.2. Analysis of Exhaled Breath

**Figure 9:**
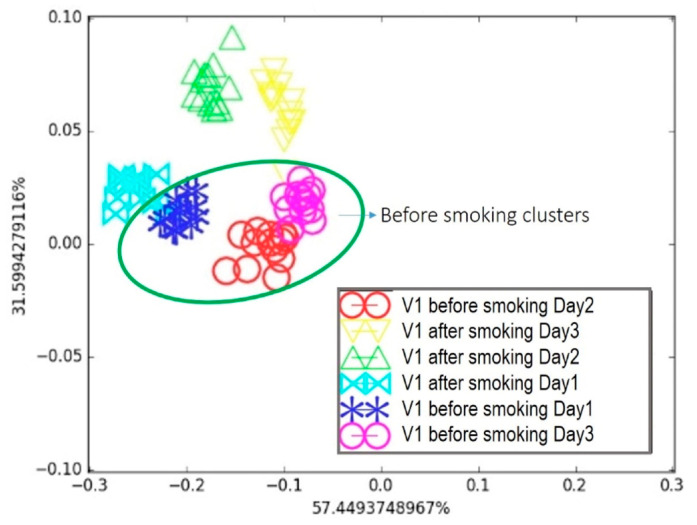
Breath testing of Volunteer 1 for three days: Comparison of breath before and after smoking on LDA breath map.

The same e-nose, assembled from the nine vQRSs selected in the previous study on cancer biomarkers, was also tested with exhaled human breath under different conditions, to move closer to healthcare monitoring applications using vQRS-based e-noses. The first volunteer (V_1_) was a smoker, and his exhaled breath was analyzed before and one hour after smoking a cigarette. This test was repeated three times on consecutive days. The resulting breath map for the first volunteer is shown in Figure 9 after an LDA treatment of the data set.

**Figure 10:**
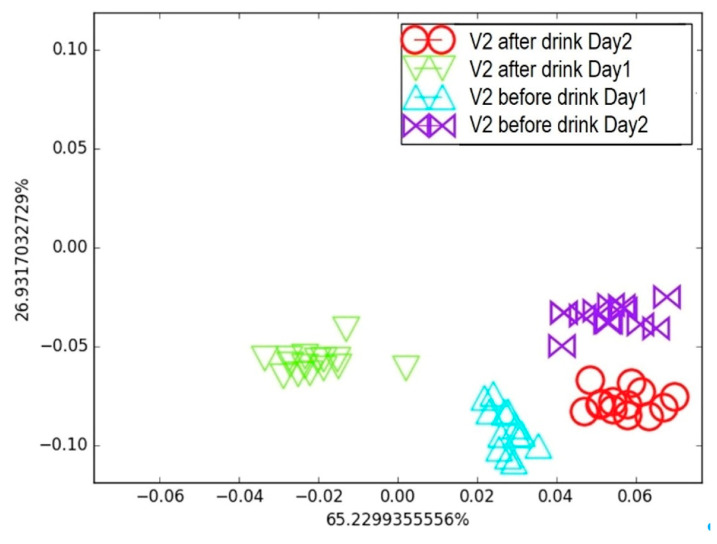
Breath testing of Volunteer 2 for two days: Comparison of breath before and after alcohol consumption on LDA breath map.

It appears in Figure 9 that, considering the day-by-day tests, the “*after smoking*” and “*before smoking*” clusters on Day 1 were separated but very close to each other. This was later attributed to the volunteer’s morning routine, which was not controlled during the first test. The breath samples were collected regardless of the volunteer’s food habits, since a person’s daily routine can affect the composition of exhaled breath, as observed in the breath map. For Day 2 and Day 3, the two “*before smoking*” clusters were in a similar area, whereas they were farther from their associated “after smoking” clusters compared to Day 1. This highlights that breath sample collection is delicate and sensitive to the volunteer’s behaviour prior to exhalation, emphasizing the need for a strict collection protocol. Nevertheless, the proposed e-nose was able to discriminate between the two samples under real conditions. The second breath condition involved alcohol consumption. The second volunteer (V_2_) provided breath samples before and after drinking a glass of wine. This test was repeated twice on consecutive days. Following the same procedure and data treatment as before, the resulting breath map is presented in Figure 10.

Interestingly, the LDA map generated from clusters of breath samples collected “*before*” and “*after*” wine consumption on Days 1 and 2 effectively discriminated the effects of alcohol and the time elapsed after consumption. The e-nose was able to detect changes in the breath samples resulting from alcohol intake; however, the “*before*” clusters did not overlap across the two consecutive days, and the same was true for the “*after*” clusters. This indicates that factors other than alcohol consumption can influence the VOC composition in exhaled breath, highlighting the need for a strict sample collection protocol combined with a well-defined training procedure. Nevertheless, these two simple experiments demonstrate that the e-nose is sufficiently sensitive to detect variations in breath prints caused by smoke and alcohol, which is very encouraging for future healthcare applications based on volatolomics.

## 4. Conclusions

This work clearly demonstrates that nanocomposite vQRS arrays, when combined with advanced data analysis techniques, represent a powerful and reliable approach for capturing and classifying olfactory signatures associated with disease biomarkers. The developed system successfully detected lung cancer-related volatile compounds at concentrations as low as 1% of the total vapor mixture while maintaining discriminative power in the presence of complex and fluctuating backgrounds.

Furthermore, the tests conducted with real human breath revealed that, despite the inherent variability of exhaled samples and the need for a carefully standardized collection protocol, the e-nose retained sufficient sensitivity to identify subtle changes induced by lifestyle factors such as smoking and alcohol consumption. These findings validate the robustness of the vQRS concept under both controlled and realistic conditions, bridging the gap between laboratory-scale proof-of-concept and potential clinical translation.

In the broader perspective, such technology offers several decisive advantages (non-invasiveness, rapid analysis, portability, and compatibility with machine-learning-based pattern recognition) that make it highly attractive for integration into next-generation healthcare systems. By enabling continuous, accessible, and early screening of patients through a simple breath test, vQRS-based e-noses could become a disruptive tool in preventive medicine, improving patient outcomes while reducing the burden on healthcare infrastructures.

## Figures and Tables

**Table 1:** e-nose composition, sensor abbreviations, and initial resistance.

*N*°	Sensor’s Name	R_0_ (kΩ)
1	PS-co-POSS/CNT	54.1
2	PMMA-co-POSS/CNT	100.5
3	PCL/CNT	19.3
4	PVP/CNT	7.4
5	PLA/CNT	3.9
6	PMMA/CNT	31.2
7	SAN/CNT	75.5
8	PS/CNT	25.9
9	PEG/CNT	10.1

**Table 2:** Composition of background vapors (**bold**) and other blends with cancer biomarkers (*italics*).

Solvent	Concentration (ppm)									
	B_0_	B_1_L	B_1_M	B_1_H	B_2_L	B_2_M	B_2_H	B_3_L	B_3_M	B_3_H
**Ethanol**	1515	1479	1444	1380	1452	1395	1292	1428	1351	1218
**Propanol**	3834	375	366	349	368	353	327	362	342	309
**Water**	1208	1180	1152	1100	1158	1112	1030	1139	1077	972
**Toluene**	331	323	315	301	317	304	282	312	295	266
**Xylene**	88	86	84	80	84	81	75	83	78	71
***Octane***	-	41	80	152	40	77	143	39	74	135
***TMB***	-	-	-	-	4.	8	15	4	8	14
***Benzaldehyde***	-	-	-	-	-	-	-	8.	16	28

## Data Availability

The original contributions presented in this study are included in the article. Further inquiries can be directed to the corresponding author.
